# Noninvasive Bioimpedance Methods From the Viewpoint of Remote Monitoring in Heart Failure

**DOI:** 10.2196/25937

**Published:** 2021-05-05

**Authors:** Pawel Krzesinski, Aleksander Sobotnicki, Adam Gacek, Janusz Siebert, Andrzej Walczak, Piotr Murawski, Grzegorz Gielerak

**Affiliations:** 1 Department of Cardiology and Internal Diseases Military Institute of Medicine Warsaw Poland; 2 Institute of Medical Technology and Equipment (ŁUKASIEWICZ-ITAM) Zabrze Poland; 3 Department of Family Medicine Medical University of Gdansk Gdansk Poland; 4 Software Engineering Department Cybernetics Faculty Military University of Technology Warsaw Poland; 5 Department of Informatics Military Institute of Medicine Warsaw Poland

**Keywords:** heart failure, impedance cardiography, remote monitoring, overhydration, hemodynamics, heart, cardiac function, cardiac, monitor

## Abstract

Heart failure (HF) is a major clinical, social, and economic problem. In view of the important role of fluid overload in the pathogenesis of HF exacerbation, early detection of fluid retention is of key importance in preventing emergency admissions for this reason. However, tools for monitoring volume status that could be widely used in the home setting are still missing. The physical properties of human tissues allow for the use of impedance-based noninvasive methods, whose different modifications are studied in patients with HF for the assessment of body hydration. The aim of this paper is to present the current state of knowledge on the possible applications of these methods for remote (home-based) monitoring of patients with HF.

## Introduction

Heart failure (HF) is a major clinical, social, and economic problem, which is attributable, among other factors, to the high frequency of exacerbations requiring urgent hospital admission. The 30-day and 6-month rates of decompensated HF are 19% to 31% and up to 50%, respectively [1-3]. In view of the important role of fluid overload in the pathogenesis of HF exacerbation, early detection of fluid retention is of key importance for preventing emergency admissions. This is theoretically possible, as there is a time window of 10 to 20 days between the onset of fluid overload and hospital admission due to decompensated HF [4]. However, tools for monitoring volume status that could be widely used in the home setting are still missing. The complexity of the pathophysiological processes involved in fluid retention and redistribution hinders the possibility of predicting exacerbation episodes with adequate sensitivity and specificity based solely on body weight monitoring [5]. This is desirable for the assessment of not only the overall fluid status, but also fluid levels in different body compartments, with a particular focus on the chest. The possible assessment of hemodynamic parameters, such as heart rate, stroke volume (SV), and cardiac output (CO), has important added value in the assessment of the etiology of cardiovascular decompensation.

The physical properties of human tissues allow for the use of impedance-based (resistance-based) noninvasive methods, whose different modifications are studied in patients with HF, for the assessment of body composition. The aim of this paper is to present the current state of knowledge on the possible applications of these methods for remote (home-based) monitoring of patients with HF.

## Impedance Cardiography

**Figure 1:**
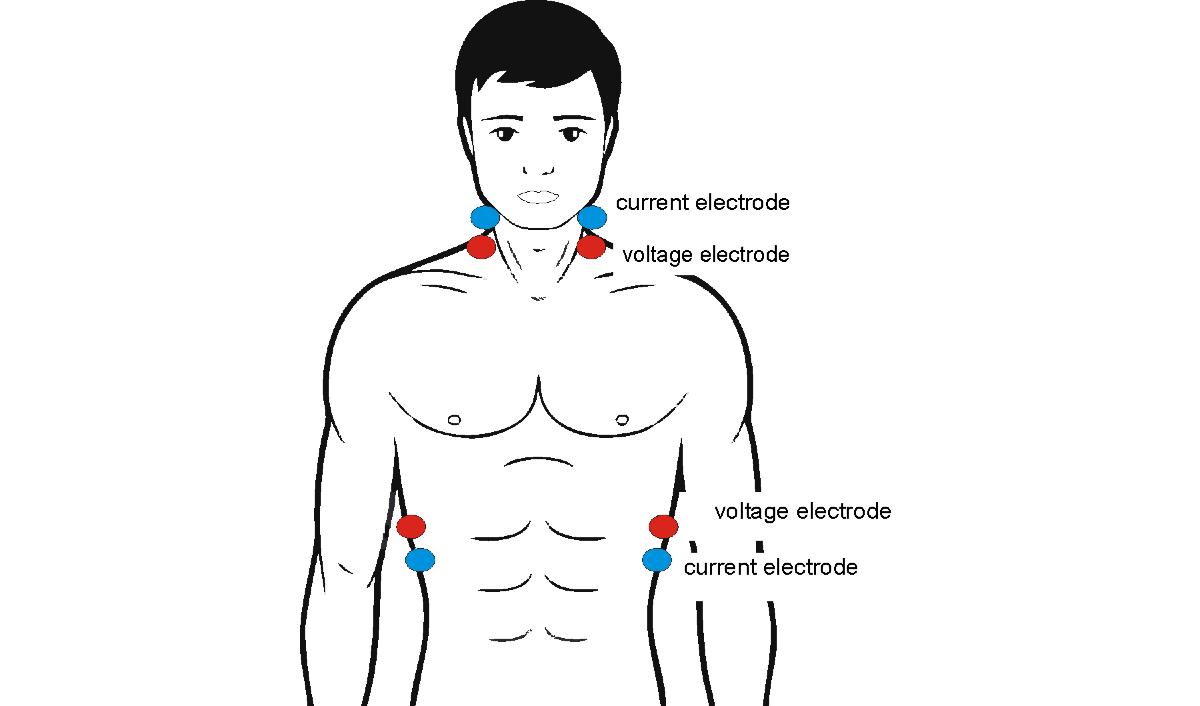
Electrode placement for tetrapolar impedance cardiography measurement.

Impedance cardiography (ICG) is a noninvasive method designed for monitoring hemodynamic parameters on the basis of analysis of thoracic electrical resistance. Eight electrodes are symmetrically placed on both sides of the neck and along the mid-axillary line (Figure 1). Electrodes supplying current (so-called current electrodes) are placed on the neck above the voltage electrodes and on the chest below the voltage electrodes. Voltage electrodes receive the potential from the thoracic region, including, among others, the heart and large vessels in the field of electric current flowing between the current electrodes. In this way, it is possible to determine thoracic fluid content [6]. During the examination, voltage changes associated with changes in blood volume and velocity in large vessels during systole and diastole are also analyzed [6]. This enables the calculation of parameters, including SV and CO [6], which is a particular advantage of the method. The estimation of SV and CO is far more difficult than the evaluation of volume status, as it requires recording reliable signals of changes in impedance due to blood flow in large vessels. The need to refer this signal to an electrocardiographic curve poses a considerable technical challenge for devices used to assess the pumping function of the heart.

ICG is also suitable for calculating parameters, including the Heather index, velocity index, and acceleration index, which are associated with the dynamics of left ventricular ejection [6]. Currently, several commercial stationary ICG devices are available, for example, Niccomo/Cardioscreen (Medis), Task Force Monitor (CNSystem), Hotman System (Hemo Sapiens Medical Inc), BioNex Impedance Cardiograph (Mindware Technologies Ltd), BioZ (CardioDynamics), NICO 100C (BIOPAC Systems), IQ2 (Noninvasive Medical Technologies Inc), and NICOM (Cheetah Medical, Inc).

The concepts of portable impedance rheographs were first proposed by researchers from the Warsaw University of Technology already more than 20 years ago. Cybulski et al [7] developed a Holter-type device prototype (ReoMonitor), which provides continuous recording of a cardiac impedance signal with simultaneous calculation of SV, left ventricular ejection time, and pre-ejection period. A comparison with echocardiography showed a high level of SV measurement consistency between these two methods (*r*=0.83). It was noted, though, that the quality of daytime recordings was lower (the percentage of properly recognized cardiac cycles was 20%-80%) compared to night-time recordings (75%-90%), which is most likely attributable to body movement and speech. Weyer et al [8] presented their concept of a mobile electrocardiographic system with multifrequency ICG assessment. The device has a wireless connection via Bluetooth, and uses a four-electrode (tetrapolar) topology identical to the one used in stationary devices. The measurements performed by the device were sent to an external module (a desktop computer). The measurements were compared with a commercial stationary device (Niccomo, Medis). A significant correlation between impedance (Z) and its time derivative (dZ/dt) was observed. The measurement of impedance resulted in a relative error below 1%, and on that basis, it was concluded that the device might be implemented in broader practice. As a possible useful solution, the application of textile electrodes for better wearing comfort was suggested. It was also noted that an active Bluetooth connection significantly increased power consumption (from 90 mW to 220 mW). To eliminate this problem, it was proposed that data transmission should not be continuous, but instead data should be stored on a memory card and sent during periods between registrations [8].

Ulbrich et al [9] developed the IMPACT Shirt (IMPedAnce Cardiography Textile) designed for SV measurement. The examination is performed by a small device recording ICG and electrocardiography (ECG) curves. The signal is transmitted via Bluetooth to a smartphone or a computer. To date, proof-of-concept studies have shown high compatibility of measurements recorded using the IMPACT Shirt and commercially available devices.

Yazdanian et al [10] also developed a concept of a portable ICG system for SV assessment, with preliminary research showing significant correlations between SV measured using the cardiac impedance method and SV determined by Doppler echocardiography (*r*=0.89, *P*<.05). Panfili et al [11] presented a device for the continuous monitoring of CO, with transmission of the recorded signal via Bluetooth. Other researchers also tended to implement ICG devices for remote monitoring to a greater or lesser extent in their scientific concepts [12-15].

**Figure 2:**
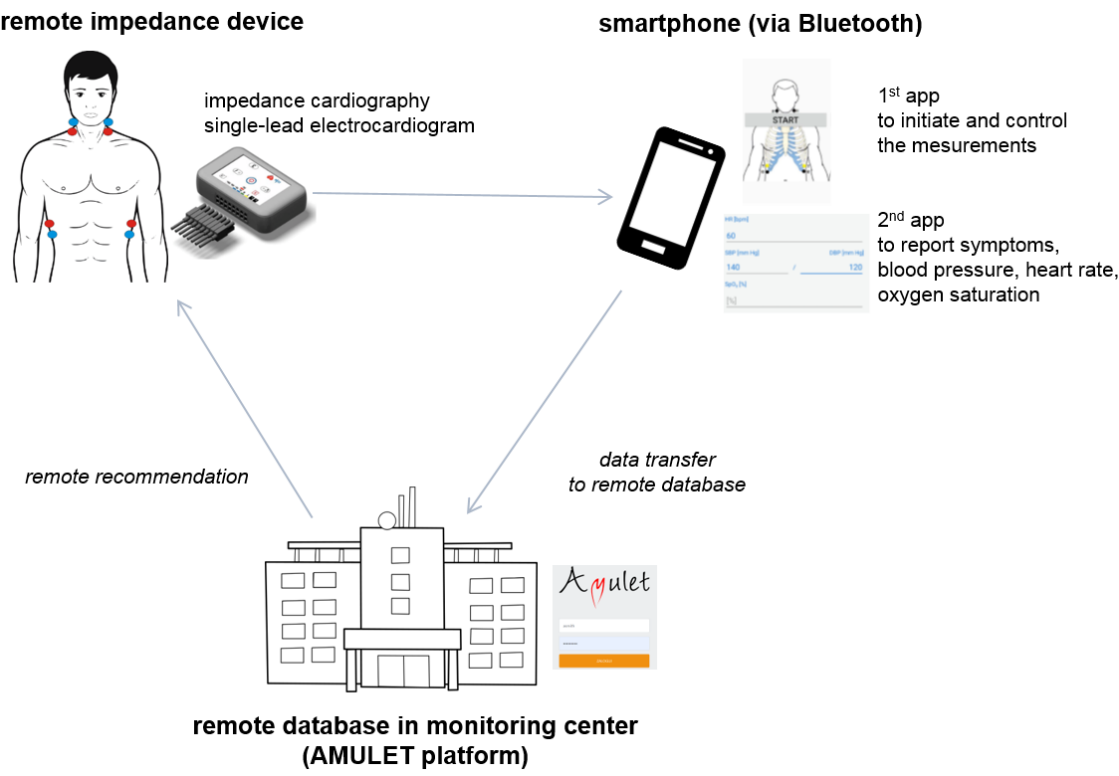
Concept of remote impedance cardiography measurements in the AMULET project.

**Figure 3:**
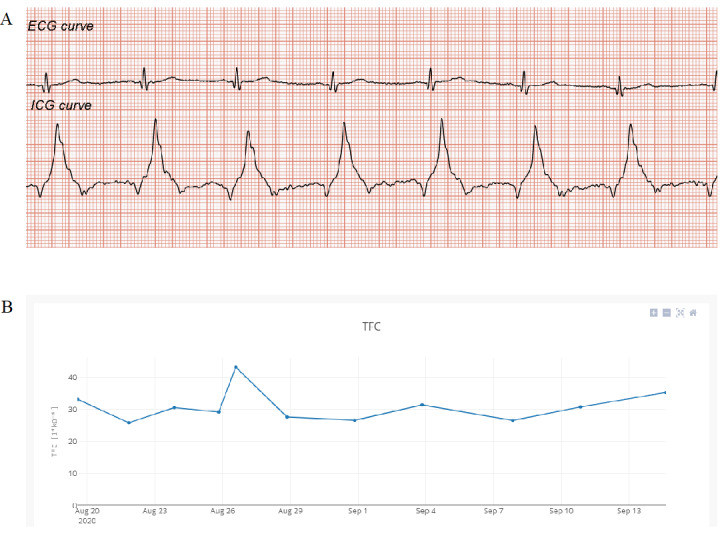
Remote database screenshots. (A) Recorded electrocardiography (ECG) and impedance cardiography (ICG) curves (the first time derivative of thoracic electrical impedance [dZ/dt]). (B) The trend of changes in thoracic fluid content (TFC) over time.

Recently, a team of researchers working on the AMULET project [16] developed a concept for remote ICG measurements with automatic data transfer to a remote electronic platform (Figure 2). Measurements are performed by a miniaturized ICG recorder measuring biological signal impedance curves and ECG, which are sent to a smartphone and then to an electronic platform, where the recorded signals are analyzed. An additional mobile app supports the reporting of symptoms experienced by the patient at the time of measurement and other vital parameters (such as blood pressure and body mass). All data are integrated and included in the patient’s individual record. There is also an option to preview recorded curves and track the trend of changes in thoracic fluid content over time (Figure 3). In addition, the system allows for sending medical recommendations based on the interpretation of recorded data to the patient. Currently, there is an ongoing pilot study assessing the feasibility of measurements and the consistency of results with a reference stationary device.

## Lung Impedance

**Figure 4:**
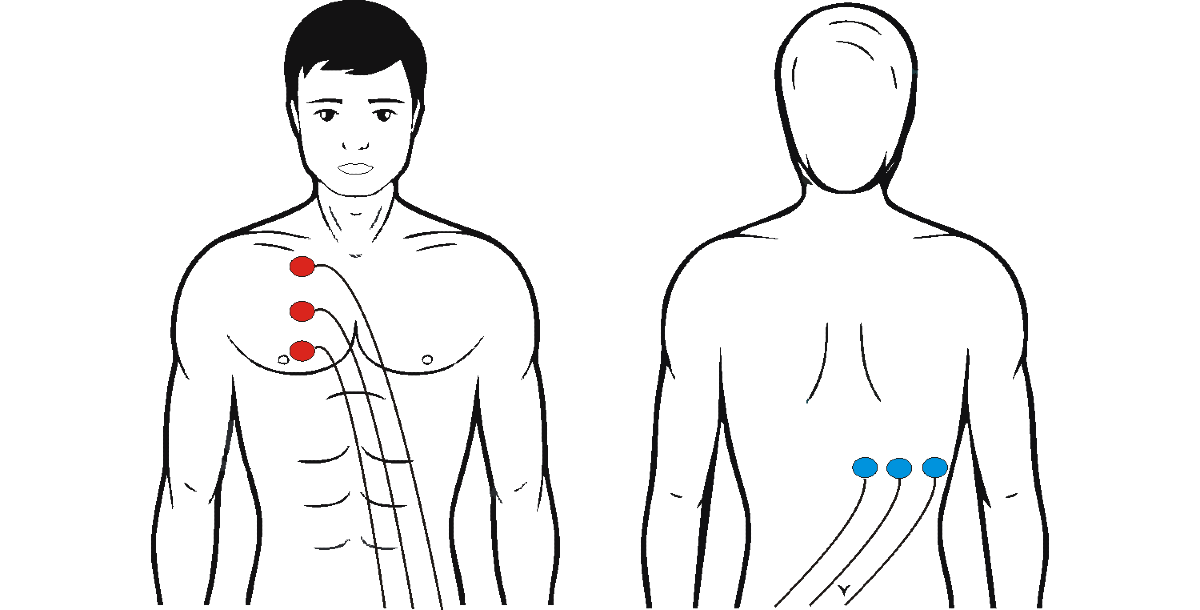
Electrode placement for lung impedance measurement.

A measurement system based on the impedance method, using modified electrode placement, was also developed (lung impedance [LI]/internal thoracic impedance [ITI]). Three electrodes are placed vertically on the front upper right side of the chest, and another three electrodes are placed on the back along the horizontal line, below the right scapula (Figure 4).

This modification is intended to eliminate the impact of large thoracic vessels on the base impedance, which is referred to as LI in this case. It was found that classical ICG measurements in the long axis were associated with a significant contribution of blood flow to chest impedance analysis, since blood conductance is 10 to 12 times higher than that of lung tissues. According to the authors' assumptions, the LI method eliminates some of these limitations, which increases its sensitivity to changes in lung fluid content. However, it should be emphasized that the applied electrode topography limits the assessment of lower lung segments, where fluid accumulation is most likely to occur first.

In 2006, a study was published [17] to report on the use of the Edema Guard Monitor (EDM, model RS-207, RS Medical Monitoring) for the assessment of the utility of ITI in predicting cardiac decompensation in patients hospitalized for cardiac reasons (acute coronary syndrome, valvular disease, or HF). The relationship between reduced ITI and the risk of pulmonary edema (confirmed clinically and radiologically) was assessed in a total of 265 patients (aged between 37 and 83 years). ITI from the first measurement was considered baseline and was used as a reference for further measurements performed every 30 minutes (for up to 24 hours or until pulmonary edema). For the purpose of the analysis, the patients were classified into the following two groups: those with pulmonary edema (DE, n=37) and those without (DNDE, n=228). ITI fluctuations in the DNDE group fell within the range of +11.0% to −10.1%. ITI deceased by over 12% in all patients in the DE group at 30 minutes (26 patients) and up to 60 minutes (11 patients) before the onset of signs of pulmonary edema. The patients presented with no symptoms of pulmonary edema and had a stable blood oxygen saturation and respiratory rate on reaching this threshold. It was concluded that a change in ITI of no more than 10% essentially excluded the risk of pulmonary edema.

These experiences were used in another study conducted in patients with ST-elevated myocardial infarction (STEMI) [18]. A hypothesis was formulated that LI-guided therapy initiated at the asymptomatic stage of pulmonary edema (Killip class I) would prevent cardiovascular destabilization and thus improve patient prognosis. The study included 560 patients with STEMI and no signs of HF on admission. During their hospitalization, repeated physical examinations, including blood pressure, heart rate, and oxygen saturation measurements, were performed. LI was monitored every 60 minutes using an impedance device (RSMM Company). Based on a pilot study, the authors selected an ITI fluctuation range from 12% to 14% as an indicator of the transition from interstitial to alveolar edema (sensitivity 96%-100%, specificity 100%). As a result, patients with LI drop of not more than 12% were included in group 1. Others were randomized to group 2 (controls) and group 3 (intervention) at a 2:1 ratio. Diuretic treatment with furosemide (oral followed by intravenous) was immediately initiated and maintained until reaching baseline LI in the intervention group. Physicians treating the control group were blinded to LI values. After discharge, the patients were followed for 6 months for the assessment of events. Of the total of 560 study patients, 347 (62%) showed a change in LI of 12% or less. None reported dyspnea, and all of them were still classified as Killip I. Of the 213 patients with LI over 12%, 142 were included in the control group (group 2) and 71 were assigned to the LI-guided therapy group (group 3). The following events were observed in groups 2 and 3: a total of 132 patients halted at Killip class IIC/III, and 10 patients presented with Killip class IV symptoms (including six deaths). In group 3, only 11% of patients developed pulmonary edema (no Killip class IV, no deaths), whereas all patients in group 2 developed pulmonary edema, including six deaths (*P*<.001). The 1-year readmission rate, 6-year mortality rate, and new-onset HF rate were lower in group 3 than in group 2.

These findings became the starting point for applying the method in outpatient care. A prospective, open-label, single-center study was conducted in a group of 222 patients with HF and left ventricular ejection fraction (LVEF) less than 35%, who were hospitalized within 12 months of recruitment with HF exacerbation [19]. Stable patients whose LI was considered reference (basal LI) were recruited. A total of 388 episodes of HF exacerbation were reported during long-term follow-up (mean 32 months). The episodes were preceded by LI decrease 2 to 4 weeks before symptom onset. One-year risk of decompensation for a 30% to 40% drop in LI was 42-fold higher compared to that for a 20% or less drop in LI, and it was more than 130 times higher for an over 40% drop. In 2016, a prospective interventional study was conducted to assess the effects of LI-guided outpatient treatment in preventing patient readmission with HF exacerbation (IMPEDANCE-HF Trial, ClinicalTrials.gov NCT01315223) [20]. A group of 252 patients after an episode of HF exacerbation (LVEF ≤35%, New York Heart Association [NYHA] II-IV) were randomized to the LI-guided treatment arm or the control arm. After 48 months (SD 32) of follow-up in the intervention group and 39 months (SD 26) of follow-up in the control group, it was found that LI-guided therapy was associated with, among other things, reduced rates of readmission caused by HF exacerbation (annual rate 0.41 vs 0.94; hazard ratio [HR] 0.63, 95% CI 0.53-0.75; *P*<.001; number needed to treat [NNT] 1.9), reduced all-cause mortality (annual rate 0.08 vs 0.14; HR 0.52, 95% CI 0.35-0.78; *P*=.002; NNT 7.5), reduced CV mortality (annual rate 0.05 vs 0.11; HR 0.41, 95% CI 0.25-0.67; *P*<.001; NNT 6.1), and reduced HF mortality (annual rate 0.03 vs 0.08; HR 0.35, 95% CI 0.15-0.58; *P*=.001; NNT 7.1). An analysis of changes in LI (ΔLIR) showed reduced ΔLIR 3 weeks prior to admission.

## Spectroscopy: Bioelectrical Impedance Analysis

Bioelectrical impedance analysis (BIA), also referred to as bioimpedance analysis, is based on the measurement of impedance (ie, electrical resistance consisting of two elements: resistance and reactance) of tissues through which low amperage electric current (≤1 mA) is passed. The phenomenon of resistance is associated with the specific resistance of different tissues, while reactance is associated with the electrical capacity of cell membranes. BIA is a method most commonly used for estimating body composition.

A group of French researchers from Institute des Nanotechnologies de Lyon developed a device for home-based wireless segmental BIA measurement. Although patients with end-stage renal disease were primarily indicated as the potential target group, the proposed solution may prove beneficial in all patients requiring regular and objective monitoring of body fluid content [21]. The device may connect to a smartphone featured with a mobile app via Bluetooth [22]. Preliminary recordings have shown that the proposed solution is sensitive to dialysis-related changes in impedance for all body compartments. However, a significant influence of body position on the results of segmental BIA has been revealed, which may lead to measurement misinterpretation, for example, during classical hemodialysis [23]. Impedance at the electrode-skin interface plays a much more important role in segmental measurements than whole-body BIA measurement. These limitations should be duly considered in the optimization of device design.

Bioimpedance spectroscopy [24], a method related to multifrequency bioimpedance, is used for home-based assessment of thoracic fluid status. It may be complementary to other approaches, for example, natriuretic peptide measurement and clinical assessment. When used in this combination, it helps to identify patients with fluid overload and worse prognosis [25]. The method enables a snap-shot home-based assessment with rapid transfer of data to the monitoring center. In their pilot study, Beckmann et al [26] showed that spectroscopic assessment (Xitron Hydra 4200, Xitron Technologies) was a valuable tool for estimating changes in body fluid volumes. When using the method to monitor five patients on diuretic therapy due to pulmonary edema, it was found that the overall assessment of whole-body and thoracic impedance allowed for reliable tracking of fluid redistribution and dehydration.

Dovancescu et al [27] developed a portable monitoring system based on a wearable fluid accumulation vest with an option to send data to a telemonitoring center via a smartphone. The vest is equipped with four textile electrodes placed pairwise on either side of the rib cage. An electronic module is placed on the back and communicates with the smartphone via Bluetooth. The system also comprises a smartphone app and an electronic database. The device enables assessment of intra- and extracellular fluid, respiratory rhythm, one-lead ECG, and patient posture and motion. A single measurement takes approximately 10 minutes, with automatic data transfer to an electronic database after completion.

An assessment of the system for predicting HF exacerbation in patients discharged after hospitalization for decompensation was planned in the SENTINEL-HF study (ClinicalTrials.gov NCT01877369) [27]. Enrollment of 180 NYHA II-IV patients hospitalized with HF in two different centers was scheduled, with patient management including daily assessment of thoracic fluid status, home visits (on days 7 and 45), telephone contact (day 14), and follow-up at endpoints (90 days). The study was launched in 2013, with the last patient recruited in April 2015. Unplanned HF-related rehospitalization along with HF exacerbation requiring increased doses of diuretics and/or associated with life quality deterioration was adopted as a composite endpoint. Decompensation alerts are triggered in the studied system based on an algorithm that tracks changes in volume status and compares them with normal variability in a given patient.

Preliminary findings from the study were presented by Darling et al [28]. The ability of patients to perform the procedures indicated in the SENTINEL-HF study and the predictive value of bioimpedance for predicting episodes of unplanned hospitalization for HF or the need to increase the dose of diuretic were assessed. Of 180 patients who consented to participate in the study, 106 completed the 75-day follow-up period; however, only 57 (53.8%) provided bioimpedance data of adequate quality. Technical factors, including noise artefacts and app malfunction, were the main reasons. Despite a relatively high predictive value of the algorithm (87% sensitivity, 70% specificity), the above-described data loss led the authors to conclude that further studies were needed to improve patient compliance and eliminate technical limitations. It should be emphasized that the device assessed in the SENTINEL study has not yet been approved by the Food and Drug Administration (FDA), and the final results of SENTINEL-HF have not been released yet.

The concept of a vest with four textile electrodes for the measurement of teletransmission was presented by Cuba-Gyllensten et al [29]. The electrodes are arranged pairwise on an elastic belt, ensuring fixation at a constant distance. The study used a prototype of the device, performing measurements at 60 different frequencies (10 kHz to 1 MHz). A number of measurements were carried out in 20 patients hospitalized with acute decompensated HF (mean age 74.7 years, mean LVEF 37%) and then correlated with clinical indicators. A significant relationship was found between changes in impedance and changes in body weight (*r*=−0.830, *P*<.001) and HF severity score (*r*=−0.537, *P*<.001). Correlations were also identified between bioimpedance measurements and LVEF (*r*=0.450, *P*=.047) and N-terminal pro-brain natriuretic peptide (*r*=−0.41, *P*=.038). The vital status of the subjects was assessed at 18 months after discharge. Three of four patients presenting with low impedance at discharge (<20 Ω) died of HF within the follow-up period. Only one death was noted among 16 other patients who left the hospital with impedance exceeding 20 Ω. The impedance values significantly differed between subjects who died and survivors, both at admission (*P*=.003) and at discharge (*P*=.02) [30].

In 2015, Seulki et al [31] presented a new wearable bioimpedance device to assess pulmonary fluid status using electrodes placed in the lower thoracic region. The idea was developed to reduce interference with other clinical equipment that is usually placed on the neck and thorax. In this system, four electrodes (two voltage and two current) are attached on the skin over the lower left thoracic region in a 2×2 array configuration with 5 cm and 12 cm center-to-center distances for the horizontal and vertical directions, respectively. Using this approach in the monitoring of patients admitted to hospital with HF decompensation, the authors revealed a good correlation of impedance changes with fluid loss (R^2^>0.80) [31]. The prognostic value of in-hospital monitoring with this wearable bioimpedance device was evaluated in a prospective study involving 36 patients (mean age 81 years, mean LVEF 45%) with acute decompensated HF [32]. At 1-year follow-up after discharge, only 12% (3/24) of patients with an increase in impedance within hospitalization died compared with 50% (6/12) of patients with an impedance decrease (*P*=.01). Although the difference for HF readmission did not reach statistical significance (17% vs 33%, *P*=.28), it was unequivocal (25% vs 75%, *P*=.01) for the composite endpoint (HF hospitalization and all-cause mortality), with a HR of 4.96 (*P*=.003) [32].

## Conclusions

Although most of the diagnostic methods discussed above remain in the stage of scientific research, they have a great potential for implementation with the use of all the advantages of telemedicine. On the other hand, multiple concerns have been raised regarding the ability of elderly patients to perform measurements at home. It turns out, however, that this factor is not necessarily a major limitation of remote monitoring. Aamodt et al [33] showed that most patients (>80%) with advanced HF were able to correctly perform home impedance measurements and send back electronic symptom questionnaires. Wireless and portable sensors, which are particularly useful for home-based monitoring, may also be an important step paving the way for these technologies [34].

The use of devices for the assessment of the volume status and hemodynamic profile in a noninvasive and patient-friendly manner should be one of the main directions in the development of telecardiology. These devices may enable systematic and frequent monitoring, which would represent a significant contribution to the complex system of care for patients with HF.

## Abbreviations

BIA: bioelectrical impedance analysis
CO: cardiac output
ECG: electrocardiography
HF: heart failure
HR: hazard ratio
ICG: impedance cardiography
ITI: internal thoracic impedance
LI: lung impedance
LVEF: left ventricular ejection fraction
NNT: number needed to treat
NYHA: New York Heart Association
STEMI: ST-elevated myocardial infarction
SV: stroke volume

## References

[1] Ponikowski P, Voors AA, Anker SD, Bueno H, Cleland JGF, Coats AJS, Falk V, González-Juanatey JR, Harjola V, Jankowska EA, Jessup M, Linde C, Nihoyannopoulos P, Parissis JT, Pieske B, Riley JP, Rosano GMC, Ruilope LM, Ruschitzka F, Rutten FH, van der Meer P, Authors/Task Force Members, Document Reviewers (2016). 2016 ESC Guidelines for the diagnosis and treatment of acute and chronic heart failure: The Task Force for the diagnosis and treatment of acute and chronic heart failure of the European Society of Cardiology (ESC). Developed with the special contribution of the Heart Failure Association (HFA) of the ESC. Eur J Heart Fail.

[2] Jencks SF, Williams MV, Coleman EA (2009). Rehospitalizations among patients in the Medicare fee-for-service program. N Engl J Med.

[3] Epstein AM, Jha AK, Orav EJ (2011). The relationship between hospital admission rates and rehospitalizations. N Engl J Med.

[4] Schiff GD, Fung S, Speroff T, McNutt RA (2003). Decompensated heart failure: symptoms, patterns of onset, and contributing factors. Am J Med.

[5] Desai AS (2012). Home monitoring heart failure care does not improve patient outcomes: looking beyond telephone-based disease management. Circulation.

[6] Krzesiński P, Gielerak G, Kowal J (2009). [Impedance cardiography - a modern tool for monitoring therapy of cardiovascular diseases]. Kardiol Pol.

[7] Cybulski G, Koźluk E, Michalak E, Niewiadomski W, Piatkowska A (2004). Holter-type impedance cardiography device. A system for continuous and non-invasive monitoring of cardiac haemodynamics. Kardiol Pol.

[8] Weyer S, Menden T, Leicht L, Leonhardt S, Wartzek T (2015). Development of a wearable multi-frequency impedance cardiography device. J Med Eng Technol.

[9] Ulbrich M, Mühlsteff J, Sipilä A, Kamppi M, Koskela A, Myry M, Wan T, Leonhardt S, Walter M (2014). The IMPACT shirt: textile integrated and portable impedance cardiography. Physiol Meas.

[10] Yazdanian H, Mahnam A, Edrisi M, Esfahani MA (2016). Design and Implementation of a Portable Impedance Cardiography System for Noninvasive Stroke Volume Monitoring. J Med Signals Sens.

[11] Panfili G, Piccini L, Maggi L, Parini S, Andreoni G (2006). A wearable device for continuous monitoring of heart mechanical function based on impedance cardiography. Conf Proc IEEE Eng Med Biol Soc.

[12] Ferreira J, Seoane F, Lindecrantz K (2013). Portable bioimpedance monitor evaluation for continuous impedance measurements. Towards wearable plethysmography applications. Annu Int Conf IEEE Eng Med Biol Soc.

[13] Nakagawara M, Yamakoshi K (2000). A portable instrument for non-invasive monitoring of beat-by-beat cardiovascular haemodynamic parameters based on the volume-compensation and electrical-admittance method. Med Biol Eng Comput.

[14] Willemsen GH, De Geus EJ, Klaver CH, Van Doornen LJ, Carroll D (1996). Ambulatory monitoring of the impedance cardiogram. Psychophysiology.

[15] Pinheiro E, Postolache O, Girão P (2013). Contactless Impedance Cardiography Using Embedded Sensors. Measurement Science Review.

[16] AMULET Project.

[17] Shochat M, Charach G, Meyler S, Meisel S, Weintraub M, Mengeritsky G, Mosseri M, Rabinovich P (2006). Prediction of cardiogenic pulmonary edema onset by monitoring right lung impedance. Intensive Care Med.

[18] Shochat M, Shotan A, Blondheim DS, Kazatsker M, Dahan I, Asif A, Shochat I, Rabinovich P, Rozenman Y, Meisel SR (2012). Usefulness of lung impedance-guided pre-emptive therapy to prevent pulmonary edema during ST-elevation myocardial infarction and to improve long-term outcomes. Am J Cardiol.

[19] Shochat M, Shotan A, Blondheim DS, Kazatsker M, Dahan I, Asif A, Shochat I, Frimerman A, Rozenman Y, Meisel SR (2015). Derivation of baseline lung impedance in chronic heart failure patients: use for monitoring pulmonary congestion and predicting admissions for decompensation. J Clin Monit Comput.

[20] Shochat MK, Shotan A, Blondheim DS, Kazatsker M, Dahan I, Asif A, Rozenman Y, Kleiner I, Weinstein JM, Frimerman A, Vasilenko L, Meisel SR (2016). Non-Invasive Lung IMPEDANCE-Guided Preemptive Treatment in Chronic Heart Failure Patients: A Randomized Controlled Trial (IMPEDANCE-HF Trial). J Card Fail.

[21] Montalibet A, Arkouche W, Bogónez Franco P, Bonnet S, Clarion A, Delhomme G, Géhin C, Gharbi S, Guillemaud R, Jallon P, Massot B, Pham P, Ribbe-Cornet E, McAdams E (2016). Design and development of an impedimetric-based system for the remote monitoring of home-based dialysis patients. Stud Health Technol Inform.

[22] Bonnet S, Bourgerette A, Gharbi S, Rubeck C, Arkouche W, Massot B, McAdams E, Montalibet A, Jallon P (2016). Wearable impedance monitoring system for dialysis patients. Annu Int Conf IEEE Eng Med Biol Soc.

[23] Montalibet A, Arkouche W, Bogonez Franco P, Bonnet S, Clarion A, Delhomme G, Gehin C, Gharbi S, Guillemaud R, Jallon P, Massot B, Pham P, Ribbe-Cornet E, McAdams E (2016). Localised impedance monitoring device for the remote clinical assessment of home-based dialysis patients. Annu Int Conf IEEE Eng Med Biol Soc.

[24] Mabote T, Wong K, Cleland JGF (2014). The utility of novel non-invasive technologies for remote hemodynamic monitoring in chronic heart failure. Expert Rev Cardiovasc Ther.

[25] Malfatto G, Corticelli A, Villani A, Giglio A, Della Rosa F, Branzi G, Facchini M, Parati G (2013). Transthoracic bioimpedance and brain natriuretic peptide assessment for prognostic stratification of outpatients with chronic systolic heart failure. Clin Cardiol.

[26] Beckmann L, Cordes A, Saygili E, Schmeink A, Schauerte P, Walter M, Leonhardt S, Dössel O, Schlegel WC (2009). Monitoring of body fluid in patients with chronic heart failure using Bioimpedance - Spectroscopy. World Congress on Medical Physics and Biomedical Engineering, September 7 - 12, 2009, Munich, Germany. IFMBE Proceedings, vol 25/7.

[27] Dovancescu S, Saczynski JS, Darling CE, Riistama J, Sert Kuniyoshi F, Meyer T, Goldberg R, McManus DD (2015). Detecting Heart Failure Decompensation by Measuring Transthoracic Bioimpedance in the Outpatient Setting: Rationale and Design of the SENTINEL-HF Study. JMIR Res Protoc.

[28] Darling CE, Dovancescu S, Saczynski JS, Riistama J, Sert Kuniyoshi F, Rock J, Meyer TE, McManus DD (2017). Bioimpedance-Based Heart Failure Deterioration Prediction Using a Prototype Fluid Accumulation Vest-Mobile Phone Dyad: An Observational Study. JMIR Cardio.

[29] Cuba-Gyllensten I, Gastelurrutia P, Riistama J, Aarts R, Nuñez J, Lupon J, Bayes-Genis A (2014). A novel wearable vest for tracking pulmonary congestion in acutely decompensated heart failure. Int J Cardiol.

[30] Gastelurrutia P, Cuba-Gyllensten I, Lupon J, Zamora E, Llibre C, Caballero Á, Riistama J, Aarts R, Bayes-Genis A (2016). Wearable vest for pulmonary congestion tracking and prognosis in heart failure: A pilot study. Int J Cardiol.

[31] Seulki L, Squillace G, Smeets C, Vandecasteele M, Grieten L, de Francisco R, Van Hoof C (2015). Congestive heart failure patient monitoring using wearable Bio-impedance sensor technology. Annu Int Conf IEEE Eng Med Biol Soc.

[32] Smeets CJP, Lee S, Groenendaal W, Squillace G, Vranken J, De Cannière H, Van Hoof C, Grieten L, Mullens W, Nijst P, Vandervoort PM (2020). The Added Value of In-Hospital Tracking of the Efficacy of Decongestion Therapy and Prognostic Value of a Wearable Thoracic Impedance Sensor in Acutely Decompensated Heart Failure With Volume Overload: Prospective Cohort Study. JMIR Cardio.

[33] Aamodt IT, Lycholip E, Celutkiene J, von Lueder T, Atar D, Falk RS, Hellesø R, Jaarsma T, Strömberg A, Lie I (2020). Self-Care Monitoring of Heart Failure Symptoms and Lung Impedance at Home Following Hospital Discharge: Longitudinal Study. J Med Internet Res.

[34] Michard F (2019). Lung water assessment: from gravimetry to wearables. J Clin Monit Comput.

